# Supplementary material for: Saccharomyces boulardii (CNCM I-745) improves intestinal damage in sepsis by remodeling intestinal flora structure
Source: Front Cell Infect Microbiol. 2025 Jul 31;15:1602792. doi: 10.3389/fcimb.2025.1602792 (PMC12350329; doi:10.3389/fcimb.2025.1602792)
Supplement: Supplementary file 1 [file DataSheet1.zip › summary/summary/8_Advanced_analyse/2_PICRUSt2_result/COG/Group.CW4_vs_CW0/PICRUSt2_COG_Group.CW4_vs_CW0_diff.pdf]

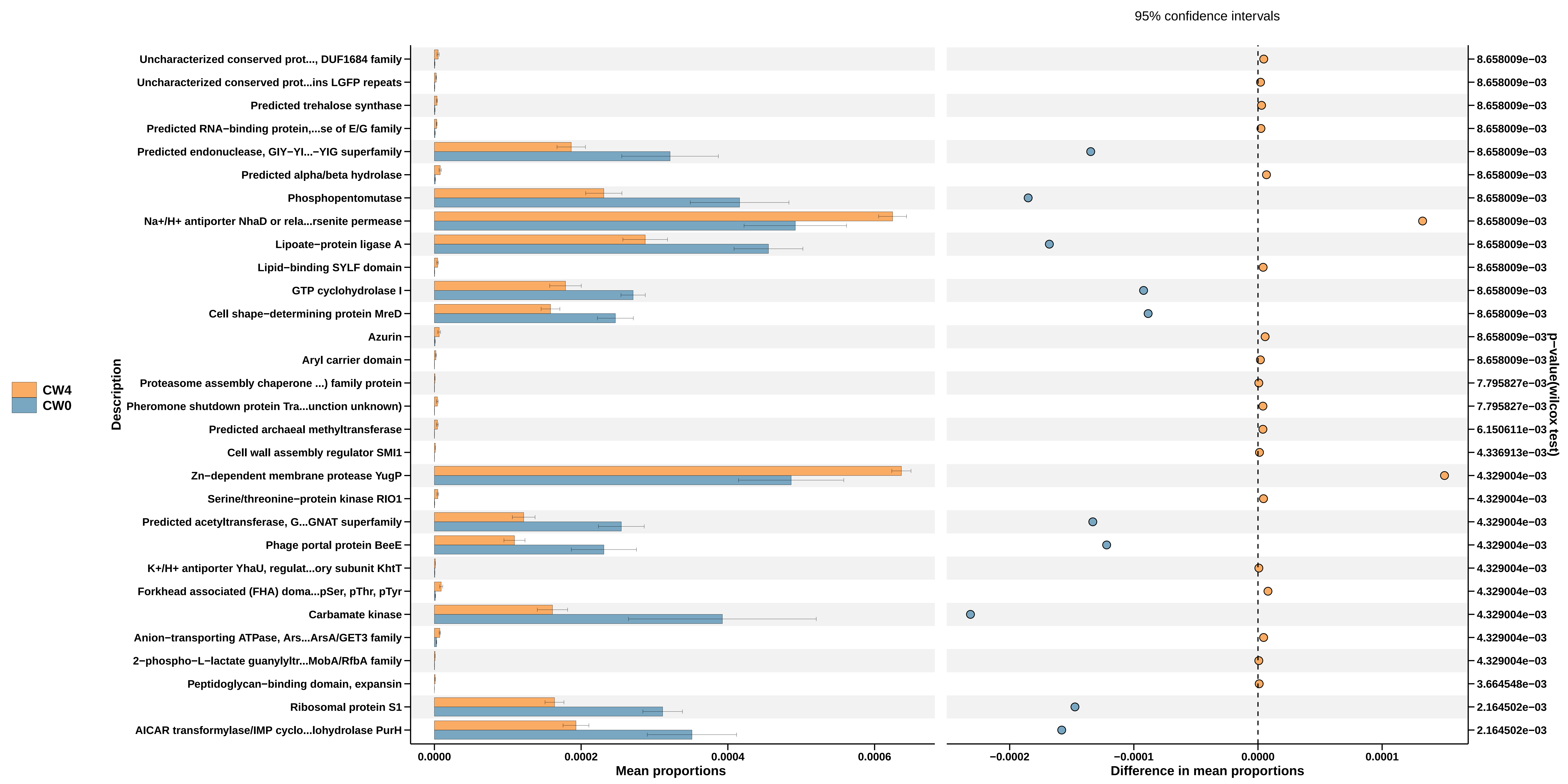

95% confidence intervals
CW4
CW0
Description
Uncharacterized conserved prot..., DUF1684 family
Uncharacterized conserved prot...ins LGFP repeats
Predicted trehalose synthase
Predicted RNA−binding protein,...se of E/G family
Predicted endonuclease, GIY−YI...−YIG superfamily
Predicted alpha/beta hydrolase
Phosphopentomutase
Na+/H+ antiporter NhaD or rela...rsenite permease
Lipoate−protein ligase A
Lipid−binding SYLF domain
GTP cyclohydrolase I
Cell shape−determining protein MreD
Azurin
Aryl carrier domain
Proteasome assembly chaperone ...) family protein
Pheromone shutdown protein Tra...unction unknown)
Predicted archaeal methyltransferase
Cell wall assembly regulator SMI1
Zn−dependent membrane protease YugP
Serine/threonine−protein kinase RIO1
Predicted acetyltransferase, G...GNAT superfamily
Phage portal protein BeeE
K+/H+ antiporter YhaU, regulat...ory subunit KhtT
Forkhead associated (FHA) doma...pSer, pThr, pTyr
Carbamate kinase
Anion−transporting ATPase, Ars...ArsA/GET3 family
2−phospho−L−lactate guanylyltr...MobA/RfbA family
Peptidoglycan−binding domain, expansin
Ribosomal protein S1
AICAR transformylase/IMP cyclo...lohydrolase PurH
0.0000
0.0002
0.0004
0.0006
Mean proportions
−0.0002
−0.0001
0.0000
0.0001
Difference in mean proportions
p−value(wilcox test)
8.658009e−03
8.658009e−03
8.658009e−03
8.658009e−03
8.658009e−03
8.658009e−03
8.658009e−03
8.658009e−03
8.658009e−03
8.658009e−03
8.658009e−03
8.658009e−03
8.658009e−03
8.658009e−03
7.795827e−03
7.795827e−03
6.150611e−03
4.336913e−03
4.329004e−03
4.329004e−03
4.329004e−03
4.329004e−03
4.329004e−03
4.329004e−03
4.329004e−03
4.329004e−03
4.329004e−03
3.664548e−03
2.164502e−03
2.164502e−03
